# Pentraxin-3-mediated complement activation in a swine model of renal ischemia/reperfusion injury

**DOI:** 10.18632/aging.202992

**Published:** 2021-04-20

**Authors:** Chiara Divella, Alessandra Stasi, Rossana Franzin, Michele Rossini, Paola Pontrelli, Fabio Sallustio, Giuseppe Stefano Netti, Elena Ranieri, Luca Lacitignola, Francesco Staffieri, Alberto Maria Crovace, Giuseppe Lucarelli, Pasquale Ditonno, Michele Battaglia, Mohamed R. Daha, Peter van der Pol, Cees van Kooten, Giuseppe Grandaliano, Loreto Gesualdo, Giovanni Stallone, Giuseppe Castellano

**Affiliations:** 1 Renal, Dialysis and Transplantation Unit, Department of Emergency and Organ Transplantation, University of Bari, Bari, Italy; 2 Department of Interdisciplinary Medicine, University of Bari, Bari, Italy; 3 Department of Basic Medical Sciences, Neuroscience and Sense Organs, University of Bari, Bari, Italy; 4 Clinical Pathology Unit, Department of Medical and Surgical Sciences, University of Foggia, Foggia, Italy; 5 Veterinary Surgery Unit, Department of Emergency and Organ Transplantation, University of Bari, Bari, Italy; 6 Urology, Andrology and Renal Transplantation Unit, Department of Emergency and Organ Transplantation, University of Bari, Bari, Italy; 7 Department of Nephrology, University of Leiden, Leiden, The Netherlands; 8 Fondazione Policlinico Universitario A. Gemelli IRCCS, Rome, Italy; 9 Nephrology, Dialysis and Transplantation Unit, Advanced Research center on Kidney Aging (A.R.K.A.), Department of Medical and Surgical Science, University of Foggia, Foggia, Italy

**Keywords:** ischemia/reperfusion injury, complement system, pentraxin 3, kidney, classical pathway

## Abstract

Pentraxins are a family of evolutionarily conserved pattern recognition molecules with pivotal roles in innate immunity and inflammation, such as opsonization of pathogens during bacterial and viral infections. In particular, the long Pentraxin 3 (PTX3) has been shown to regulate several aspects of vascular and tissue inflammation during solid organ transplantation.

Our study investigated the role of PTX3 as possible modulator of Complement activation in a swine model of renal ischemia/reperfusion (I/R) injury.

We demonstrated that I/R injury induced early PTX3 deposits at peritubular and glomerular capillary levels. Confocal laser scanning microscopy revealed PTX3 deposits co-localizing with CD31^+^ endothelial cells. In addition, PTX3 was associated with infiltrating macrophages (CD163), dendritic cells (SWC3a) and myofibroblasts (FSP1). In particular, we demonstrated a significant PTX3-mediated activation of classical (C1q-mediated) and lectin (MBL-mediated) pathways of Complement. Interestingly, PTX3 deposits co-localized with activation of the terminal Complement complex (C5b-9) on endothelial cells, indicating that PTX3-mediated Complement activation occurred mainly at the renal vascular level. In conclusion, these data indicate that PTX3 might be a potential therapeutic target to prevent Complement-induced I/R injury.

## INTRODUCTION

Ischemia-reperfusion (I/R) injury represents the major cause of acute kidney injury after transplantation and is characterized by a significant activation of the complement system [1, 2]. In this scenario, endothelial cells (EC) play a critical role in the maladaptive repair after I/R, leading to early fibrosis by endothelial to mesenchymal transition (EndMT) [3]. During the reperfusion phase, Complement orchestrates immunological and inflammatory processes, contributing to various immune and inflammatory diseases [2–5]. Other essential components of the humoral arm of the innate immune system are represented by pentraxins that are thought to play a pivotal role in vascular biology [6].

Pentraxins are a family of multimeric soluble proteins [6] that are classified into short and long families based on their structure [7]. These evolutionarily conserved proteins are acute-phase effectors, that serve as a sensors for inflammation initiation and rapidly increased in plasma during an infection [8]. The long pentraxin 3 (PTX3) is a soluble pattern recognition molecule that is crucial in innate immune protection and can activate complement system [9–11].

In particular, PTX3 induced classical and lectin pathway activation by binding with C1q, MBL, Ficolin-2 and is able to affect the alternative pathway via CFH [10–12].

In contrast with other liver-produced pentraxins in the bloodstream (i.e. CRP), PTX3 can be released by resident cells within the site of inflammation, for example from mononuclear phagocytes, dendritic cells, fibroblasts, and EC [9] acting in a paracrine manner [13]. PTX3 is also stored in a ready-made form in neutrophils, localized in specific granules, and secreted in response to the recognition of microbial moieties [14]. In EC, the expression of PTX3 is readily induced by TNF-α and IL-1β, giving a transition from a quiescent, anti-inflammatory phenotype, to a procoagulant and proinflammatory state, thereby strongly regulating the microvascular function [7, 8]. For that reason, PTX3 levels has been described as an indicator of disease activity at sites of inflammation. In chronic kidney disease, the increase in protein levels of PTX3 has been correlated with GFR declines and cardiovascular complications, however, little is still known of the role of PTX3 in early setting as I/R-induced acute kidney injury [15, 16].

The role of PTX3 in renal inflammatory diseases is bivalent, from a side the protein can activate classical and lectin pathways promoting initial inflammation and injury [10–12]. From the other side, the N-terminal domain of PTX3 modulated complement activation, attenuated leukocyte recruitment and inhibited interstitial fibrosis in acute renal injury promoting tissue repair [17–20].

Complement plays a pivotal role in the pathophysiology of I/R injury-induced acute kidney injury [21, 22]. In a swine model of renal I/R injury, we demonstrated the pivotal role of Complement system activation in inducing EndMT and early fibrosis, with the involvement of both classical and lectin pathways [23]. Moreover, we demonstrated that therapeutic inhibition of these complement pathways by recombinant C1-INH (rhC1INH) produced a significant reduction in complement deposition, with decreased recruitment of infiltrating inflammatory cells and tubulointerstitial damage [23]. These results were also confirmed by Delpech PO et al [24]; a significant modulation in C1q, MASP and C4d glomerular and tubular deposition was assessed after 30 min post-reperfusion indicating a central role of C1-INH to counteract classical and lectin pathways.

In this study, we investigated the possible involvement of PTX3 in mediating early Complement activation in renal I/R injury, characterizing the different cellular sources of PTX3.

## RESULTS

### PTX3 is expressed by endothelial cells and immune infiltrating cells in a swine model of I/R injury

**Figure 1:**
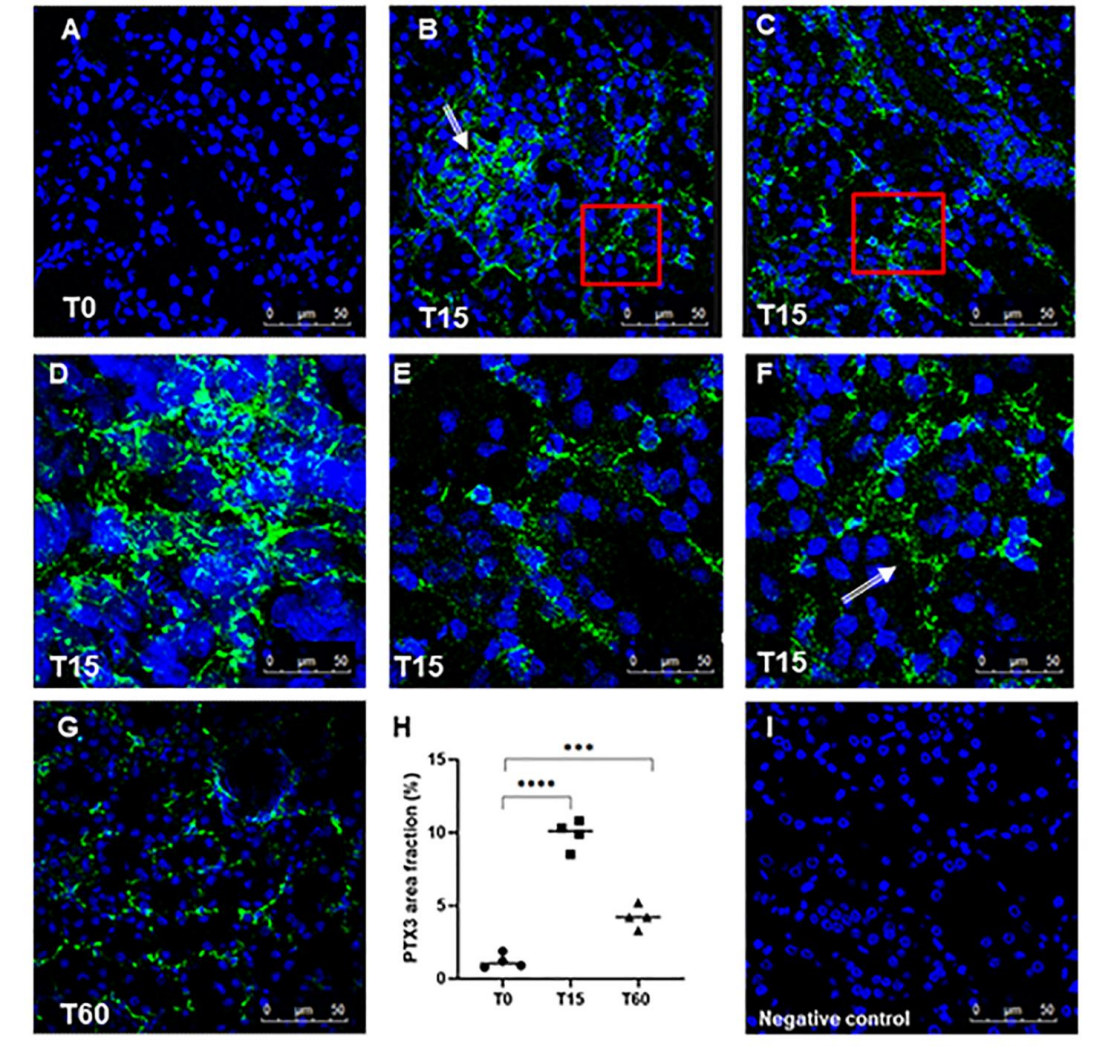
**Analysis of PTX3 deposits in a swine model of I/R injury.** Indirect immunofluorescence for PTX3 was performed on frozen pig kidney sections. A limited presence of PTX3 was observed in the biopsies at T0 (**A**). PTX3 deposits were observed after 15 min of reperfusion (**B**, **C**) at interstitial (**E**, zoomed image), peri-tubular (**F**, zoomed image) and glomerular (**D**) capillary levels. After 60 min the PTX3 deposits were still described at the level of peritubular capillaries (**G**). (**I**) Negative staining control for immunofluorescence was performed on cryosections with irrelevant primary antibodies for experimental conditions. Nuclei were highlighted with TO-PRO 3 in blue. Magnification 630X. (**H**) Quantification of PTX3 demonstrated a statistically significant increase after 15 min of reperfusion compared to basal biopsies. Results were expressed as % ± s.d. of positive area /high power field (hpf). *p<0.05 versus T0.

First, we investigated the presence of PTX3 in a swine model of warm I/R-induced renal injury. We observed very limited PTX3 deposits in normal tissue (Figure 1A). I/R injury caused a diffuse deposition of PTX3 already at 15min following reperfusion (Figure 1B, 1C) in the tubulo-interstitial area (Figure 1E), at peritubular capillaries (Figure 1D; arrow) and at glomerular levels (Figure 1D). PTX3 deposits were still detectable 1 hour after reperfusion at the level of peritubular capillaries (Figure 1G). In our previous work [23] we demonstrated that the main features of I/R injury are tubular epithelial cell apoptosis and the recruitment of infiltrating inflammatory cells as monocytes, dendritic cells and lymphocytes. Nevertheless, by routine histological evaluation (Supplementary Figure 1), we demonstrated that 30 min of warm ischemia followed by 15 min of reperfusion induced early tubule-interstitial damage, characterized by bigger capillary congestion and focal cytoplasmic vacuolation of renal tubule epithelium, compared to basal condition.

**Figure 2:**
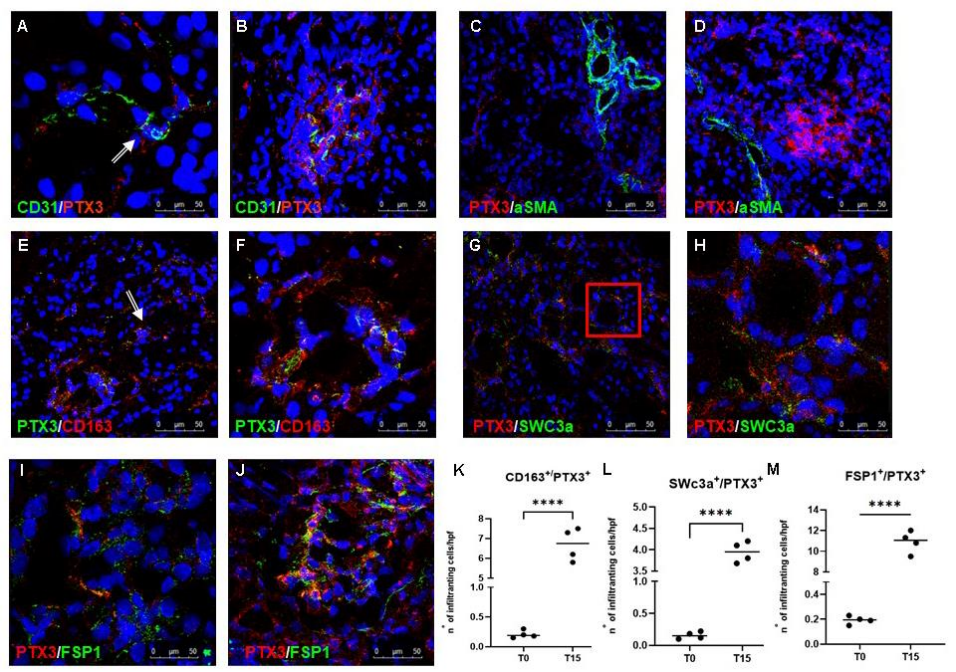
**Characterization of the PTX3-associated cellular pattern in I/R injury.** Frozen pig kidney sections were analyzed by indirect immunofluorescence to characterize the PTX3 source after 15 min of reperfusion. Co-localization between CD31 and PTX3 on renal EC was evident (**A**, **B** yellow staining). Activated myofibroblasts identified by alpha-smooth muscle actin (green) were negative for PTX3 (red; α-SMA^+^/PTX3^-^, **C**, **D**). Monocytes/macrophages identified by CD163 (red) co-localized with PTX3 (green; CD163^+^/PTX3^+^ yellow, (**E**) particular of **E**, **F**). Dendritic cells identified by SWC3a (green) were intensively positive for PTX3 (red; SWC3a^+^/PTX3^+^ yellow, (**G**) particular of **G**, **H**). Myofibroblasts identified by fibroblast-specific protein 1 (FSP1, red) co-localized with PTX3 (green; FSP1^+^/PTX3^+^ yellow, **I**, **J**). Nuclei were highlighted with TO-PRO 3 in blue. Original magnifications were x630. Quantification of CD163^+^/PTX3^+^ (**K**), SWC3a^+^/PTX3^+^ (**L**) and FSP1^+^/PTX3^+^ (**M**) cells demonstrated a statistically significant increase after 15 min of reperfusion compared to basal biopsies. Results were expressed as mean ± s.d. of infiltrating cells/high power field (hpf). *p<0.05 versus T0.

To further characterize the cellular localization of PTX3 deposits and evaluate its potential effect in the modulation of inflammatory response and injury, we performed double-immunostaining and confocal microscopy analysis. PTX3 protein expression was detected in most of the EC at peritubular (Figure 2A) and glomerular (Figure 2B) capillary levels, 15 min after reperfusion.

It is well known that I/R injury is characterized by increased activation of innate and adaptive immune responses, including inflammatory cell trafficking into the diseased organ that further exacerbates injury via immune cells and Complement system [25]. In our swine model, we also observed, already 15 min after reperfusion, a dense inflammatory infiltrate composed largely of macrophages and dendritic cells in the tubule-interstitial area. We found that both these antigen-presenting cells were characterized by increased PTX3 expression when compared to T0, since we observed an increased number of CD163^+^/PTX3^+^ (Figure 2E, 2F, 2K) and SWC3a^+^/PTX3^+^ (Figure 2G, 2H, 2L) cells at tubule interstitial levels at T15.

### PTX3 expression can contribute to EndMT in I/R injury

In previous observations, we demonstrated that I/R injury was responsible for EndMT [26, 27], characterized by the acquisition of a mesenchymal phenotype by EC with the loss of specific endothelial markers and the gain of mesenchymal markers, such as fibroblast-specific protein 1 (FSP-1), neuronal cadherin (N-cadherin) and alpha-smooth muscle actin (alpha-SMA). Thus, we investigated whether PTX3 expression by EC could affect this process. As expected, when we investigated alpha-SMA expression, as markers of activated myofibroblast, we did not find any co-localization between alpha-SMA and PTX3 (Figure 2C, 2D). On the contrary, we observed an increase in tubulo-interstitial FSP1^+^/PTX3^+^myofibroblasts throughout the observation period (Figure 2K–2M).

### PTX3 deposits are associated with activation of the complement system

**Figure 3:**
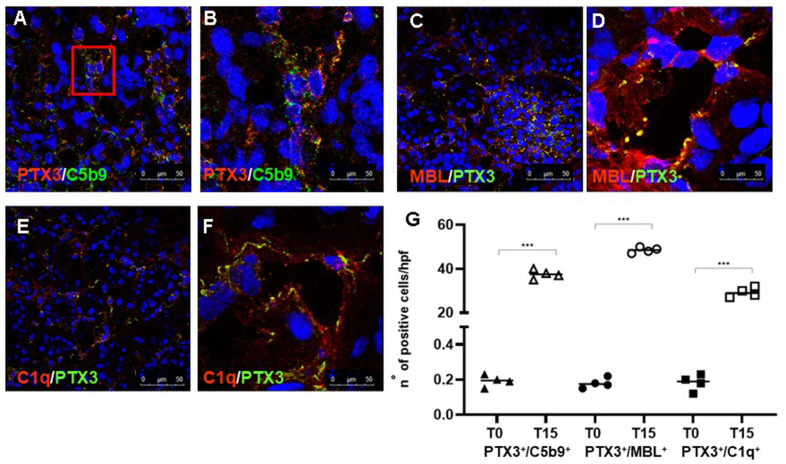
**PTX3-mediated Complement activation in a pig model of I/R injury.** Frozen pig kidney sections were examined by indirect immunofluorescence to investigate the co-localization (yellow staining) of C5b9 (green) and PTX3 (red) deposits (**A**, **B**). The co-localization between PTX3 (green) with MBL (red, **C**, **D**) and C1q (**E**, **F**) was investigated by immunofluorescence/confocal microscopy. PTX3 co-localized with MBL (**C**, **D**, yellow staining) and C1q (**E**, **F**, merge) at peri-glomerular (**E**) and peri-tubular (**D**, **F**) capillary sites. In confocal microscopy images nuclei were stained with TO-PRO 3 (blue). (**G**) Quantification of C5b9^+^/PTX3^+^, MBL^+^/PTX3^+^and C1q^+^/PTX3^+^cells compared to basal biopsies. Results were expressed as % ± s.d. of positive area /high power field (hpf). *p<0.05 versus T0.

Finally, we investigated whether PTX3 deposits were associated with Complement activation. Indeed, as other components of the pentraxin family, PTX3 can regulate the activation of classical Complement pathway [7]. To define the relationship between PTX3 and Complement activation, we performed a double-label immunofluorescence to evaluate the expression of PTX3 and the terminal Complement complex, C5b-9, using an antibody directed against a C9-neo-epitope. We observed a significant co-localization of PTX3 and C5b-9 deposits (Figure 3A, 3B). The Complement terminal complex was localized at the peritubular level as well as within the peritubular capillaries along the endothelial cell layer, as we previously demonstrated [23, 28]. Since PTX3 can activate the Complement system through the classic and lectin pathways, we evaluated the deposition of C1q and MBL in renal parenchyma. Interestingly, C1q (Figure 3E, 3F) and MBL (Figure 3C, 3D) deposits were mainly found at the interstitial and capillary level (Figure 3C through 3F), as previously described [23] and colocalized with PTX3 deposits.

### C1-inhibitor interferes with PTX3 binding on endothelial cells

**Figure 4:**
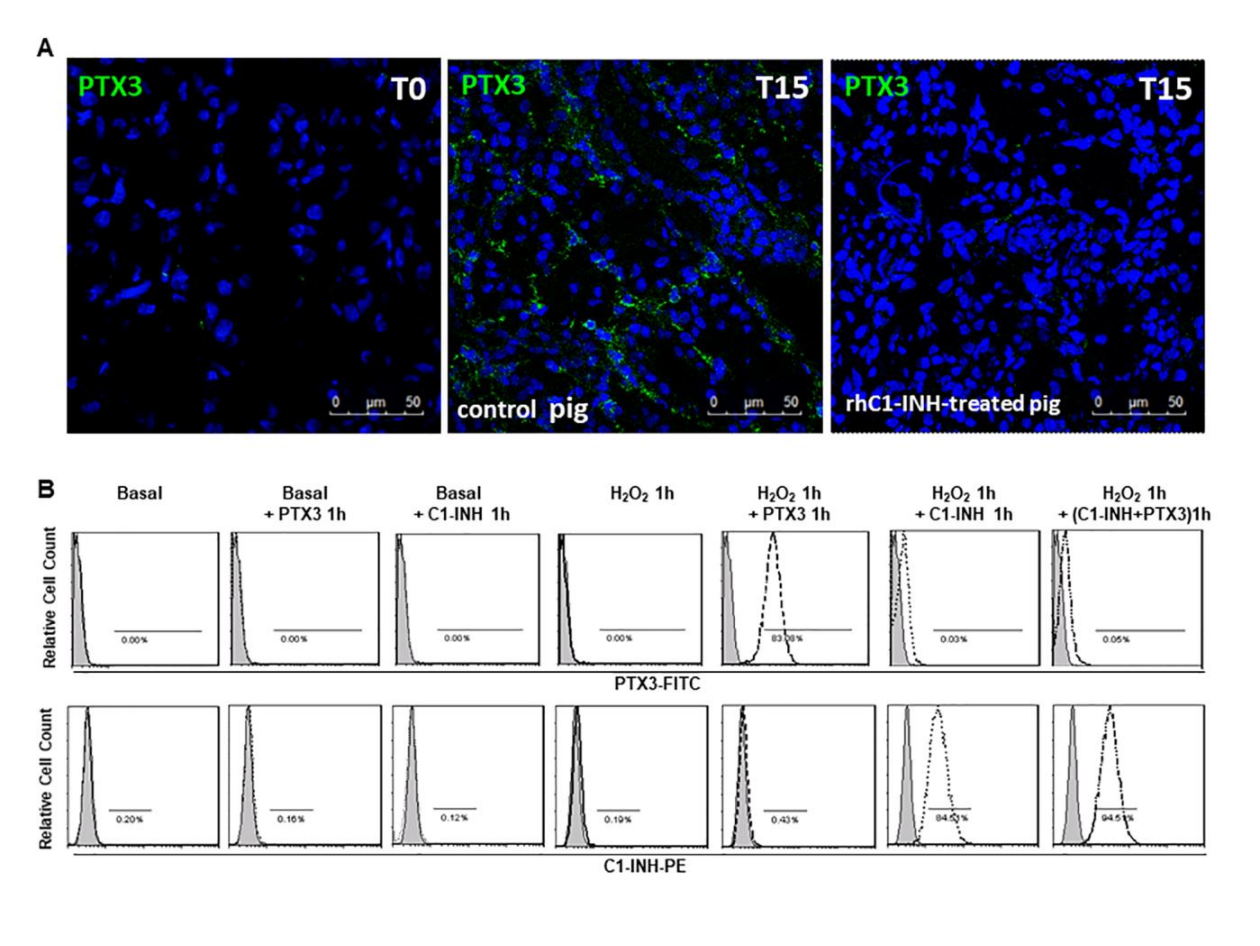
**C1-inhibitor prevents PTX-3 binding on endothelial cells.** (**A**) Frozen pig kidney sections were analyzed by indirect immunofluorescence to characterize the PTX3 source after 15 min of reperfusion in control and rhC1-INH treated pigs. PTX3 deposits were observed at the level of peritubular capillaries in control pigs. rhC1-INH infusion prevented PTX3 deposits on ECs. (**B**) FACS showed that ECs in basal condition did not bind both rhC1-INH and PTX3. Both PTX3 and rhC1-INH presented a significant binding on H2O2-activated ECs. When H2O2-activated EC were co-stimulated with PTX3 and rhC1-INH, rhC1INH prevented PTX3 binding. Results are representative of three independent experiments.

In our previous work [23] we demonstrated that C1-inhibitor administration led to significant reduction in complement deposition, with decreased recruitment of infiltrating inflammatory cells and tubulointerstitial damage. Therefore, we examined the level of PTX3 expression in rhC1-INH treated animals. We found that the infusion of C1-inhibitor reduced PTX3 deposits at peritubular capillaries and interstitial level after 15 min post-reperfusion (Figure 4A).

Moreover, to support the hypothesis that the reduction of PTX3 deposits in rhC1-INH treated animals was associated with the inhibition of endothelial damage, we performed *in vitro* experiments and we evaluated rhC1-INH and PTX3 binding on cultured EC under normal conditions or in the presence of cellular stress (Figure 4B). FACS analysis showed that EC in normal condition did not bind both rhC1-INH and PTX3. In accordance with our previous study [26] we observed an increased cellular binding of rhC1-INH on H_2_O_2_-stimulated EC compared to basal condition. Moreover, in the absence of rhC1-INH, PTX3 could bind activated EC. Interestingly, when H2O2-activated EC were incubated with PTX3 and rhC1-INH, we observed that C1INH was able to protect EC upon blocking PTX3 binding.

## DISCUSSION

In this study we demonstrated PTX3 deposition in the early phase of renal I/R injury and its possible contribution in the development of EndMT. Interestingly, we found that PTX3-mediated Complement activation occurs mainly at vascular level, co-localizing with C1q and MBL, the recognition molecules of classical and lectin pathways of Complement cascade.

I/R injury triggers a marked inflammatory response characterized by Complement activation, oxygen free radicals and proinflammatory cytokine production, resulting in activation of vascular endothelium and peripheral leucocytes [29, 30]. During I/R injury, Complement activation leads to complement components deposition on the surface membrane of damaged and dysfunctional EC, with the simultaneous generation of anaphylatoxins and the amplification of inflammatory process [31]. During inflammation, PTX3 increases rapidly and could exert a central role in modulating endothelial response. Indeed, PTX3 has been indicated as a potential biomarker of vascular endothelial dysfunction in several diseases, including chronic kidney disease, preeclampsia and several vascular diseases [7, 16, 17, 32]. We also demonstrated that PTX3 is involved in other vascular complications such as the failure of arteriovenous fistula in hemodialysis patients [33]. These observations suggest that PTX3 could be a bridge between inflammatory response and endothelial dysfunction [34]. In line with these studies, we observed PTX3 deposits at endothelial level already after 15 min following reperfusion (Figure 2A, 2B). Our results also demonstrated that in the early phase of I/R injury, PTX3 colocalized with myofibroblast marker, FSP-1 (Figure 2I, 2J) but not with alpha-SMA, marker expressed by activated myofibroblast (Figure 2C, 2D). Taken together, these data could suggest that endothelial dysfunction and EndMT process [35], observed in I/R animals [26], firstly occurred in EC expressing PTX3.

The link between PTX3 and inflammatory cells is widely recognized. In this paper, we specifically focused on the inherent effects of PTX3 in interstitial infiltration of leucocytes that are a major source of PTX3 [36]. In particular, we found macrophages and dendritic cells, after 15 min following reperfusion, expressing higher levels of PTX3 (Figure 2E through 2H). These data are in agreement with the increasing body of evidence suggesting a relevant role for innate immunity in mediating early damage in I/R injury [23]. An early activation of Complement in renal tissue after I/R injury leads to the generation of several inflammatory mediators that increase the recruitment of immune cells [21, 23, 37, 38]. Recent studies have identified PTX3 as one of the principal components of the network that orchestrates the inflammatory response triggered by I/R injury [39]. In different experimental models of I/R injury, PTX3 can exert dual opposite roles on specific tissues [40, 41]. Early production of PTX3 is associated with renal damage, since it induces early expression of endothelial adhesion molecule and chemokines that accelerate local maladaptive inflammatory response.

On the contrary, the prolonged local production of PTX3 prevents excessive organ inflammation and dysfunction [41].

PTX3 is a Complement cascade modulator [9, 42]; this is in agreement with pleiotropic properties of PTX3 indicating a dual role of PTX3 as a modulator or amplifier of the innate immune response [39]. Initially, PTX3 activates Complement by binding C1q and MBL [43]; however, early increased inflammation needs to be limited to the target area. Therefore, PTX3, by recruiting factor H or inhibiting angiogenesis, could also reduce the inflammatory response and complement activation preserving renal parenchyma from inflammatory damage [43].

Although Complement activation in I/R in rodents is mainly localized at tubular level [44], during the reperfusion phase, the endothelium is the primary target of different pro-inflammatory agents, including Complement mediators [2]. We previously demonstrated that in swine model of I/R injury as well as in DGF patients, the activation of the Complement system occurs in the early phase, on peritubular capillaries, within the interstitium, and on the glomerular endothelium [23]. Our data showed a clear co-localization of C5b-9 deposits on PTX3^+^EC after 15 min following reperfusion (Figure 3A, 3B). Therefore, renal endothelium seems to be the prevalent site of PTX3-mediated Complement activation in the early phase of I/R injury in both preclinical and clinical settings.

The interaction of pentraxins with C1q and its role in the activation of the classical Complement pathway are well described [45–47]. In the context of the innate immune responses, PTX3 can bind different Complement components and modulate Complement activation [43, 48]. PTX3 activates Complement by C1q binding [49]. Our results in animal model clearly demonstrated that PTX3 might mediate classical pathway activation by interacting with C1q (Figure 3E, 3F). Moreover, PTX3 also modulates the lectin pathway of the Complement, as shown in Figure 3C, 3D. MBL binds PTX3 via its collagen-like domain [45] and MBL/PTX3 complexes recruit C1q and elicit C3 and C4 deposition on target cell surfaces.

All together, these results suggest the central role of PTX3 in mediating kidney damage in I/R injury, that could have important implications for Complement-directed therapies in renal I/R injury.

In our previous study [26], we investigated the involvement of complement in mediating EC activation by using a recombinant form of C1-INH, a potent inhibitor of proteases of the classical and lectin complement pathways (C1r, C1 s and MASP2). In the same animal model, we showed (Figure 4A) that therapeutic inhibition of both pathways by rhC1INH reduced PTX3 deposits at peritubular capillaries and interstitial level after 15 min following reperfusion. These data confirmed with *in vitro* results on EC (Figure 4B), led us to hypothesize that the rhC1INH might protect damaged EC upon blocking PTX3 binding. In literature, there are evidence about the binding of C1-INH to endothelial adhesion molecules, expressed on activated endothelium, called selectins, in particular P and E-selectins [50, 51]. This binding on EC can interfere with endothelial-leukocyte interaction during inflammation and it represents another important anti-inflammatory mechanism [50, 51]. Therefore, we hypothesized that rh-C1INH can bind activated EC and mediates local regulation of complement activation and inflammatory process. In our previous studies, we have demonstrated the involvement of complement in I/R injury and other immune mediated renal disease [38, 52–54]. The mechanisms of Complement activation in this animal model could have important implications for the interpretation of data expected in the human setting. To successfully develop therapeutic interventions targeted towards Complement-activation [36, 54], it is essential to establish the validity of pig data relative to what occurs in clinical circumstances. Since this research is limited to observational studies, further experiments are needed to delineate the interconnected mechanisms between PTX3 and Complement that might highlight new therapeutic strategies. From the results above, our data support the hypothesis that PTX3 might regulate multiple aspects of Complement-mediated I/R injury thereby representing a potential therapeutic target.

## MATERIALS AND METHODS

### Renal I/R injury pig model

The animal model of renal I/R injury was developed as previously described [23]. After approval by the ethical committee of the Ministry of Health, 4-month-old female Large White pigs (n=8, n=4 for group, 20 kg)underwent experimental open surgical procedure under general anesthesia. The animals were fasted for 24 hours before the induction of anesthesia. The electrocardiogram, heart rate, hemoglobin saturation of oxygen, respiratory gas composition, respiratory rate, tidal volume, airway pressure, systolic arterial blood pressure, and central venous pressure were continuously monitored and recorded automatically (Ohmeda Modulus CD; DatexOhmeda, Helsinki, Finland). The left renal artery and vein were isolated and a vessel loop was positioned around the renal artery with a right-angle clamp. A renal biopsy was performed before ischemia (T0). Then, the ischemic phase was induced (30 min) by pulling on the vessel loop. Multiple biopsies were then performed at 15, 30, and 60 min after reperfusion; animals were sacrificed 24 hours after the surgical procedure. A portion of each biopsy specimen was immediately snap frozen in optimal cutting temperature (Tissuetek, Pittsburgh, PA) medium and stored in liquid nitrogen. Another portion was fixed in buffered formalin (4%) for 12 hours and embedded in paraffin using standard procedures.

### Microscopy study

Paraffin-embedded renal specimens from renal biopsies were used for conventional histological staining (H&E, periodic acid-Schiff). Images were acquired by Aperio ScanScope CS2 device (Aperio Technologies, Vista, CA, USA). Tubule-interstitial and glomerular lesions were evaluated using a qualitative analysis by two observers (C.D., M.R.) who were unaware of the origin of the slides.

### Antibodies

The primary antibodies used in this study recognized the following antigens: PTX3 (MNB4: direct against PTX3 N-terminal domain, Exira Life Sciences In., Larsen, Switzerland);CD163 (monocytes/macrophages, US biological, Swampscott, MA); SWC3a (dendritic cells, [55] 74-22-15A, BD Biosciences); FSP1 (fibroblast specific protein 1, Abcam, Cambridge, UK);alpha-smooth muscle actin (Santa Cruz Biotechnology Inc.; Santa Cruz, CA, USA); C1q (R9/2, AbDSerotec; Kidlington, United Kingdom); MBL (3E7: direct against MBL carbohydrate recognition domain, Hycult biotechnology, Uden, the Netherlands) and C9 neo antigen (aE11, Hycult biotechnology). The cross reactivity was validated by pre-incubating the specific antibodies, before their use, with human peptides used to raise them. The pre-incubation abolished specific staining on swine tissue.

### Tissue immunofluorescence and confocal laser scanning microscopy

The characterization and localization of PTX3 signal were investigated on frozen tissue included in OCT medium (Tissuetek). The slides were incubated with 5% rabbit serum for 1 hour at 37° C. Slides were then incubated for 1 hour at room temperature with specific antibodies. After three washes in PBS, slides were then incubated with the appropriate secondary antibodies(Alexa Flour 488 and 555, Molecular Probes, Eugene, OR). All sections were counterstained with TO-PRO-3 (Molecular Probes). Negative controls were prepared with irrelevant antibodies. The sections were analyzed using the Leica TCS SP2 (Leica, Wetzlar, Germany) confocal laser-scanning microscope. The number of infiltrating cells was measured in at least10 high power (x630) fields/section by two independent observers blinded to the origin of the slides. The final counts were the mean of the two measures. In no case interobserver variability was higher than 20%.

### Cell culture and flow cytometry analysis

Human umbilical vein endothelial cells (HUVEC, EC) were purchased from American Type Culture Collection (ATCC-LGC Standards, Sesto San Giovanni, Italy). EC were grown in their recommended media, EndoGro (Merck Millipore, Darmstadt, Germany). EC were plated at a density of 10,000cells/cm^2^ and were stimulated with H2O2 treatment (3%, 1 hour). Then basal and stimulated EC were washed twice with PBS and were removed with PBS-EDTA 2mM and trypsin 0.001×. Then cells were resuspended in PBS and were incubated respectively with PTX3 (recombinant human PTX3, Sigma-Aldrich, Merck, Germany) (1ug/ml) or/and with rhC1-INH (Ruconest^®^, Pharming) (2.5ug/ml) for 60 min. After washing three times with PBS 1X, cells were resuspended in flow cytometry (FACS) buffer (phosphate-buffered saline, pH 7.2, 0.2% bovine serum albumin, and 0.02% sodium azide) and incubated with FCR blocking reagent (Miltenyi Biotec) for 10 min at room temperature. After blocking, ECs were incubated with rabbit anti-human C1-INH (provided by Prof. M. Daha, University of Leiden, 1//100 dilution) or/and with rat anti-PTX3 (MNB4, Exira Life Sciences In., 1/20 dilution) at room temperature for 30 min and washed with the FACS buffer. Then, cells were incubated with goat anti-rabbit IgG PE (Molecular Probes, 1/100 dilution) or/with anti-rat IgG FITC (Molecular Probes, 1/100 dilution) at room temperature for 30 min and washed three times. Cells were analyzed with FC500 (Beckman Coulter, Brea, CA, USA) and Kaluza software. The area of positivity was determined by using an isotype-matched mAb, and, in total, 104 events for each sample were acquired. Three independent experiments were performed.

### Statistical analysis

Data are presented as mean ± standard deviation (SD) and are compared using analysis of variance or paired Student t-test, as appropriate. Differences were considered statistically significant when p values were less than 0.05. Data were analyzed using the Statview software package (5.0 version)(SAS Inc. Co., Cary, NC, USA). Graphs were displayed using GraphPad Prism Software 5.

## Supplementary Material

Supplementary Figure 1
