# Supplementary material for: Pentraxin-3-mediated complement activation in a swine model of renal ischemia/reperfusion injury
Source: Aging (Albany NY). 2021 Apr 20;13(8):10920–33. doi: 10.18632/aging.202992 (PMC8109140; doi:10.18632/aging.202992)
Supplement: Supplementary Figure 1 [file aging-13-202992-s001.pdf]

SUPPLEMENTARY FIGURE

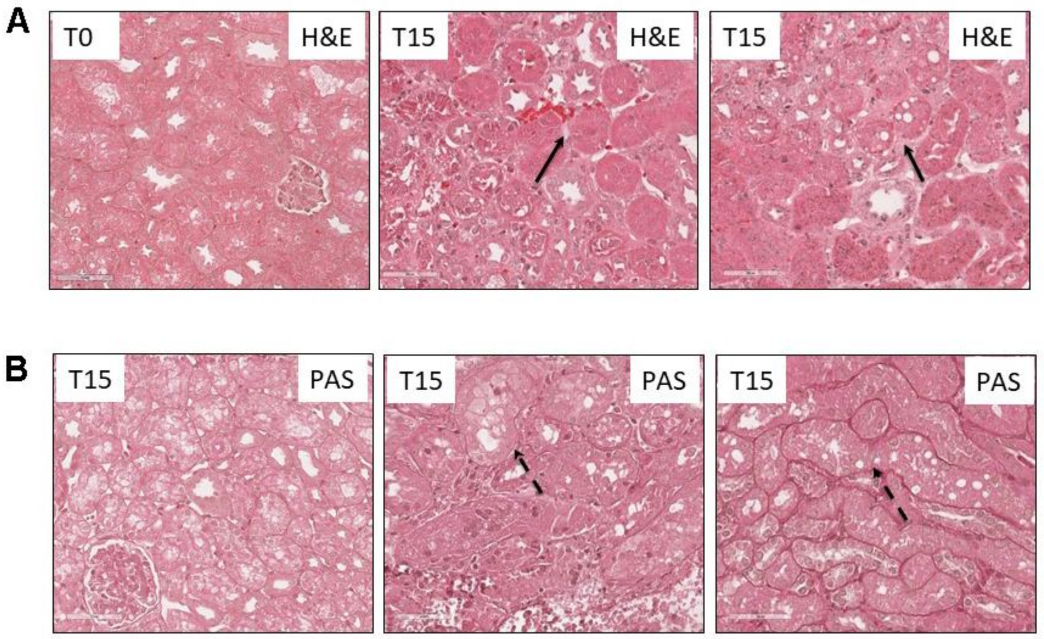

Supplementary Figure 1. Modulation of early tubulointerstitial damage in a swine model of I/R injury. Compared with basal conditions, 30 min of warm ischemia followed by 15 min of reperfusion induced capillary congestion (**A**, black arrows, H&E staining) and focal vacuolization at the tubulointerstitial level (**B**, dotted arrows, periodic acid-Schiff (PAS) staining). Magnification 20X.
